# Thermoanalytical Investigation of Some Sulfone-Containing Drugs

**DOI:** 10.1155/2012/439082

**Published:** 2012-06-20

**Authors:** Nahla N. Salama, Mohammed A. El Ries, Safaa Toubar, Maha Abd El Hamid, Mohammed I. Walash

**Affiliations:** ^1^Pharmaceutical Chemistry Department, National Organization for Drug Control and Research, Pyramids Avenue, P.O. Box 29, Giza, Egypt; ^2^Analytical Chemistry Department, Faculty of Pharmacy, Helwan University, Cairo 1860, Egypt; ^3^Analytical Chemistry Department, Faculty of Pharmacy, Mansoura University, Mansoura 35516, Egypt

## Abstract

The thermal behavior of some sulfone-containing drugs, namely, dapsone (DDS), dimethylsulfone (MSM), and topiramate (TOP) in drug substances, and products were investigated using different thermal techniques. These include thermogravimetry (TGA), derivative thermogravimetry (DTG), differential thermal analysis (DTA), and differential scanning calorimetry (DSC). The thermogravimetric data allowed the determination of the kinetic parameters: activation energy (*E*_*a*_), frequency factor (*A*), and reaction order (*n*). The thermal degradation of dapsone and topiramate was followed a first-order kinetic behavior. The calculated data evidenced a zero-order kinetic for dimethylsulfone. The relative thermal stabilities of the studied drugs have been evaluated and follow the order DDS > TOP > MSM. The purity was determined using DSC for the studied compounds, in drug substances and products. The results were in agreement with the recommended pharmacopoeia and manufacturer methods. DSC curves obtained from the tablets suggest compatibility between the drugs, excipients and/or coformulated drugs. The fragmentation pathway of dapsone with mass spectrometry was taken as example, to correlate the thermal decomposition with the resulted MS-EI. The decomposition modes were investigated, and the possible fragmentation pathways were suggested by mass spectrometry.

## 1. Introduction

**Dapsone (DDS)**

It is antibacterial drug used in the treatment of *Mycobacterium leprae* infection (leprosy), and malaria [1, 2]. It is official in BP and USP [3, 4]:

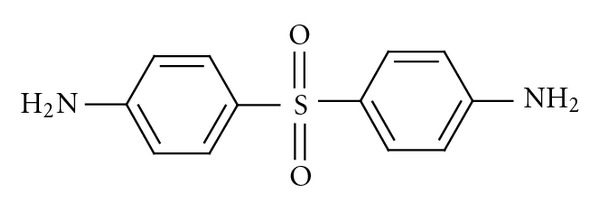

**Dimethyl Sulfone (MSM)**

It is used as anti-inflammatory agent [5, 6] and in combination with glucosamine and chondroitin to treat or prevent osteoarthritis [7, 8]:
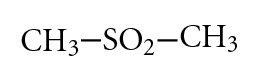

**Topiramate (TOP)**

It is antiepileptic drug [9]. It is official in USP [4]:

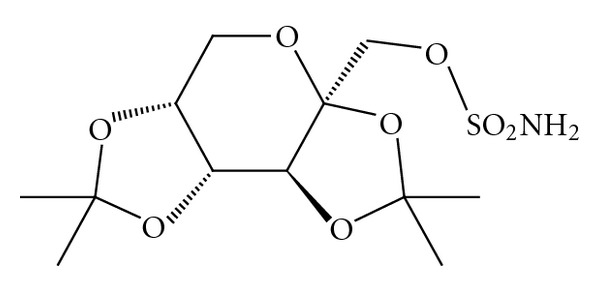


Different analytical methods were reported for the assay of DDS in dosage forms and in biological fluids, including spectroscopy [10–21], electrochemical methods [22, 23], and chromatography [24–28]. In literature two GC methods were reported for determination of dimethyl sulfone [29, 30]. Topiramate has no ultraviolet, visible, or fluorescence absorption, and available methods for analysis of the drug in biological fluids and pharmaceutical dosage formulation consisted of gas chromatography (GC) coupled with flame ionization (FID) or nitrogen phosphorous detection (NPD) [31–33] and fluorescence polarization immunoassay [34]. HPLC methods, including ionic chromatography, or using refractive index (RI), chemiluminescent nitrogen, or MS detector are described [35–37]. Analysis of the drug in human plasma following derivatization with 9-fluorenylmethyl chloroformate (FMOC-Cl) or 4-chloro-7-nitrobenzofurazan (NBD-Cl) using fluorescence or UV detection has been reported [38–41]. 

Thermal analysis is a group of techniques in which a physical property of a substance and/or its reaction products is measured as a function of temperature whilst the substance is subjected to a controlled temperature program. These methods find widespread use in quality control of drugs, with a view to improvement of the final product and for the determination of drug quality via the technological parameters [42]. These techniques include thermogravimetry (TGA), derivative thermogravimetry (DTG), differential thermal analysis (DTA), and differential scanning calorimetric (DSC) methods. In a thermogravimetric analysis the mass of a sample in a controlled atmosphere is recorded as a function of temperature or time as the temperature of the sample is increased [43]. TGA is commonly employed in research and testing to determine degradation temperatures, absorbed moisture content of materials, decomposition and kinetic parameters. Differential thermal analysis (DTA) is a thermoanalytical technique, in which the temperature between the material under study and an inert reference (Δ*T*) is measured as a function of temperature (*T*), while the substance and reference material are subjected to temperature program [44]. A plot of Δ*T* against time or temperature is called DTA curve or thermogram. Changes in the sample, either exothermic or endothermic, can be detected relative to the inert reference. DTA curve can be used only as a finger print for identification purposes but usually the applications of this method are the determination of phase diagrams, heat change measurements, and decomposition in various atmospheres [45, 46]. Differential scanning calorimetry (DSC) is a thermoanalytical technique in which the difference in the amount of heat required to increase the temperature of a sample and reference is measured as a function of temperature. DSC is used in the pharmaceutical industry as analytical tool of great importance for the identification and purity testing of active drugs, yielding results rapidly and efficiently [47]. It is also applied for the quality control of raw materials used in pharmaceutical products [48].

Mass spectrometry is used to elucidate the structure of compounds. The compound is ionized and fragmented using the electron spray ionization technique. The term “decomposition” or “degradation” signifies the breakdown of one or more constituents of the substance into simple atomic groups. The thermal decomposition of a solid may also involve physical transformation such as melting and sublimation, and these changes may exert a significant effect on the subsequent chemical reaction [49].

 In modern analytical laboratory, there is always a need for rapid and significant methods for identification and purity determination of drugs. The determination of the melting point using DSC method has been satisfactorily used as a method of evaluating the degree of purity of a compounds.

In literature no references have been found for application of TGA/DTG, DTA, and DSC for thermal decomposition of DDS, MSM, and TOP in their drug substances and products. Therefore, the objective of this study was to investigate the thermal stability, kinetic parameters, and compatibility between the studied drugs and excipients and/or coformulated drugs.

## 2. Experimental

### 2.1. Materials

Dapsone was kindly supplied from The Nile Company for Pharmaceuticals & Chemical Industries, Cairo; its purity was found to be 99.66% according to the official method [3]. Dapsone tablets labeled to contain 50 mg Dapsone/tablet—The Nile Company for pharmaceuticals & Chemical Industries, Cairo (batch no. 16226)—were purchased from local market. Dimethyl sulfone was obtained from Eva Pharma for Pharmaceuticals & Medical Applicances S.A.E. Co., Egypt; its purity was found to be 99.00% according to the manufacturer GC method. MSM tablets labeled to contain 1000 mg dimethyl sulfone/tablet (batch no: 702180) were purchased from local market. Genuphil tablets were obtained from Eva Pharma for Pharmaceuticals & Medical Applicances S.A.E. Co., Egypt. Each tablet contains MSM 375 mg, chondroitin sulphate 300 mg, and glucosamine sulphate 375 mg (batch no. 908458), purchased from the market. Topiramate was supplied from Delta Pharma, Egypt; its purity was found to be 99.00% according to the USP method [4]. Topamax 25 tablets labeled to contain 25 mg TOP/tablet Janssen—Cilag Co. (batch no. 9FS1Q00).

### 2.2. Instrumentation and Methods:

Thermogravimetry, Derivative Thermogravimetry (TGA/DTG), and Differential Thermal Analysis (DTA). TGA/DTG, and DTA curves of drug substances were recorded using simultaneous Shimadzu thermogravimetric analyzer TGA-60 H with TA 60 software in dry nitrogen atmosphere at a flow rate of 30 mL/min in platinum crucible with an empty platinum crucible as a reference. The experiments were performed from ambient temperature up to 1000°C with a heating rate of 10°C/min. The sample mass was about 5 mg of the drug without any further treatment.

The kinetic parameters of decomposition such as activation energy (*E*_*a*_), frequency factor (*A*), and reaction order (*n*) were calculated from TGA/DTG curves. Arrhenius equation [46] and the mathematical models of Horowitz and Metzger [47] and Coats and Redfern [50] were used for kinetic parameters determination.

Differential Scanning Salorimetry (DSC). The DSC curves of Dapsone, dimethyl sulfone and Topiramate were recorded using Shimadzu-DSC 50, in dynamic nitrogen atmosphere with a constant flow rate of 30 mL/min and heating rate of 2°C/min, up to temperature 200°C/min using a mass of about 2 mg of sample packed in platinum pan. DSC equipment was preliminarily calibrated with standard reference of indium. Ten tablets or capsules of the drug products of the studied drugs were homogenized, and accurately weighed amount equivalent to 2 mg of each drug substance was packed in the pan. Then the DSC curves were recorded.

Mass Spectrometry Electron Impact (MS-EI). Mass spectra of Dapsone were recorded using the Shimadzu-GC-MS-QP 1000 EX quadruple mass spectrometer with Electron Impact detector equipped with GC-MS data system. 

Melting point instrumentas OptiMelt Automated Melting Point System, *SRS* Stanford Research System.

## 3. Results and Discussion

### 3.1. Thermal Characterization of the Investigated Compounds

**Dapsone**

**Figure 1:**
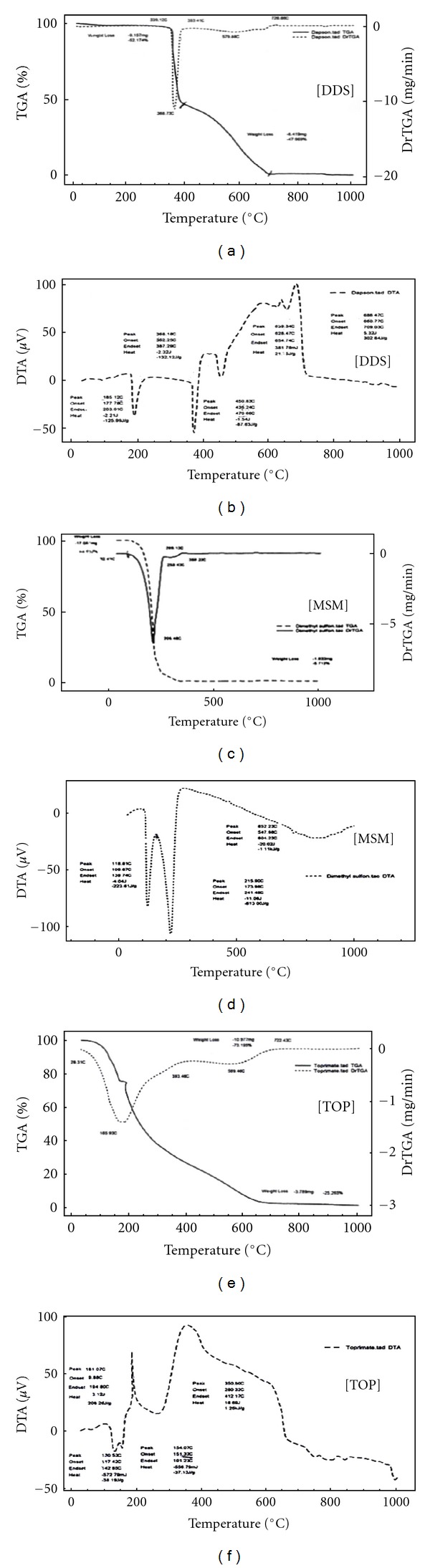
Thermal decomposition TGA/DTG and DTA for Dapsone, dimethyl sulfone, and Topiramate.

The TGA/DTG curves of DDS presented in Figure 1 revealed two thermal decomposition stages and thermal stability up to 339°C. The first step shows a mass loss (Δ*m* = 52.00%) in the interval of 339.12–393. 41°C, suggesting the release of C_6_H_6_SO molecule (51.00%, calc.). The second decomposition step shows a mass loss (Δ*m* = 48.00%) in the temperature range 393. 41–726.86°C, suggesting the loss of C_6_H_6_N_2_O molecule (49.00%, calc.) by cleavage of amino group. The results are presented in Table 1.

The DTA curve (Figure 1) exhibits endothermic and exothermic peaks. The first endothermic peak at 185.12°C is due to the melting of the compound. The sharp endothermic peak at 368.18°C is attributed to the first decomposition corresponding to the first mass loss observed in TGA/DTG thermogram curves as shown in Figure 1.

**Scheme 1:**
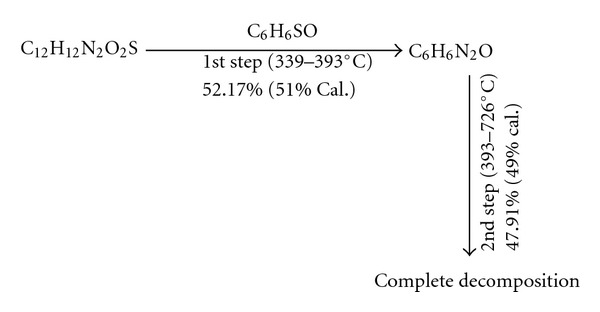
The suggested thermal degradation of Dapsone.

The sharp exothermic peaks at (639.34–686°C) are due to the pyrolysis of the compound (Table 2). The suggested thermal decomposition pathway of Dapsone is summarized in Scheme 1.

**Dimethyl Sulfone**

TGA/DTG and DTA plots of MSM are represented in Figure 1. The thermal behavior of MSM shows complete mass loss after the melting point as shown in Figure 1. The DTG plot contained a large sharp peak, which abruptly returned to zero baseline this peak is characteristic of the zero-order kinetic process of evaporation. The mass loss was determined, and the results were stated in Table 1.

The DTA plot exhibited two endotherms corresponding to melting at 118°C, and the second endothermic peak suggests vapor pressure of the molten sample between the melting and boiling points.

**Topiramate**

The TGA/DTG and DTA curves are shown in Figure 1. The TGA/DTG curves show that TOP is thermally stable up to 151.32°C. TGA/DTG curves show three thermal decomposition steps. The first step shows mass losses 73.46% in temperature range (151.32–393.46°C) corresponding to formation of carbonaceous residue. The second and third steps show mass loss 25.265% in temperature range (393.46–722.43°C), due to pyrolysis of carbonaceous residue (Table 1).

The DTA curve presented in Figure 1 exhibits endothermic and exothermic peaks. The first peak at 130.53°C is due to melting of compound. The sharp endothermic peak at 154.07°C is attributed to the first decomposition corresponding to the first mass loss observed in TGA/DTG curves. The broad exothermic peaks at 181.07°C, 350.8°C are due to the pyrolysis of the compound (Table 1).

### 3.2. Kinetic Analysis

The kinetics of the main thermal decomposition steps of DDS, TOP, and MSM were studied using the Arrhenius equation [46]. Computation of the kinetic parameters was based on the use of the Arrhenius equation applied to the solid-state reactions. The logarithmic form of the Arrhenius equation is

(1) $$\;\ln K=\ln A-\frac{E}{\text{RT}}.$$

The Arrhenius equation can be combined with the rate equation, which is written as

(2) $$\frac{d\alpha }{dt}=K\left(T\right)f\left(\alpha \right).$$

Combining (1) and (2) gives the following relation:

(3) $$\ln\left[\frac{\text{d}\alpha /\text{d}t}{f\left(\alpha \right)}\right]=\ln A-\frac{E}{\text{RT}},$$

where (*α*) is the decomposed fraction, (d*α*/d*t*) is the rate of the reaction, *f*(*α*) is a function of the actual composition of the sample, *K* is the specific rate constant, (*A*) is the pre- exponential term, *E* is the activation energy, *R*: gas constant, and *T*: temperature in degrees Kelvin. Alfa (*α*) (the fraction reacted at a particular temperature) was calculated from the weight of the sample at temperature *T*(*W*_*t*_), the initial weight (*Wi*), and the final weight (*W*_*f*_) using the following equation:

(4) $$\;\alpha \;=\frac{Wi-W_{T}}{Wi-W_{f}}.$$

The differential form of (4) gives

(5) $$\frac{d\alpha }{dt}\;=\;\frac{{dw}_{t}/dt}{Wi-W_{f}}.$$

The function (*dw*_*t*_/*dt*)  is obtained from the DTG data. Then, the rate of the reaction can be calculated directly. This value of (*dw*_*t*_/*dt*)  obtained from (5) is substituted into (4), and finally, a plot of ln⁡⁡[(*dα*/*dt*)/*f*(*α*)]  versus 1/*T* is constructed. The activation energy and the preexponential terms were calculated from the slope and the intercept, respectively [46].

The mathematical models used for identifying the term *f*(*α*), which refer to the order of reaction, were calculated according to reference table [51, 52]. The kinetic parameters obtained from the first decomposition step were activation energy (*E*_*a*_), frequency factor (*A*), reaction order (*n*), and correlation coefficient (*R*). The calculated data evidenced a first-order kinetics behavior for DDS and TOP, with *E*_*a*_ value 485.83 KJ  mol^−1^ and 93.78 KJ  mol^−1^ respectively, while for MSM the data evidence zero-order *E*_*a*_, with *E*_*a*_ value 51.003 KJ  mol^−1^ (Table 2). 

The kinetic studies of the main thermal decomposition steps of DDS and TOP were investigated also by using mathematical models of Horowitz and Metzger [47] and Coats-Redfern, respectively [50]. The calculated data evidenced also a first-order kinetics behavior for DDS with *E*_*a*_ values 485 kJ  mol^−1^ (HM) and 456  KJ mol^−1^ (CR) and frequency factor 1.27 × 10^32^ Sec^−1^ (CR). Also the calculated data evidenced a first-order kinetics behavior for TOP with *E*_*a*_ values 136.76 kJ  mol^−1^ (HM) and 144.43  KJ mol^−1^ (CR) and frequency factor 7.6 × 10^8^  Sec^−1^ (Table 3):

(6) $$\log\cdot \left[\log\frac{W_{f}}{W_{f}-W}\right]=\frac{\theta \cdot E^{*}}{2.303\;RT_{s}^{2}}-\log2.303.$$

where *W* is the mass loss at time *t* and *W*_*f*_ after total decomposition, *R* is the gas constant, *T*_*s*_ is the DTG peak temperature and *θ* = *T* − *T*_*s*_. A plot of log⁡⁡[log⁡⁡*W*_*f*_/(*W*_*f*_ − *W*)]  *versus* *θ* will give a straight line, and *E*_*a*_ was then calculated from the slope (Table 3):

(7) $$\begin{array}{cc} \log\left(\frac{\log\left[W_{f}/W_{f}-W\right]}{T^{2}}\right) & =\log\left[\frac{\text{AR}}{\phi E^{*}}\left(1-\frac{2\text{RT}}{E^{*}}\right)\right]\\ & \;-\frac{E^{*}}{2.303\text{RT}} \end{array}$$

*ϕ* is the heating rate (°C /min). Since 1−2RT/E*≅ 1, the plot of the left-hand side of (7) *versus *1000/*T* will give a straight line. *E*_*a*_ was then calculated from the slope and the frequency factor (*A*) was obtained from the intercept (Table 3).

### 3.3. Correlation between the Mass Spectra and Thermal Behavior of Dapsone

**Scheme 2:**
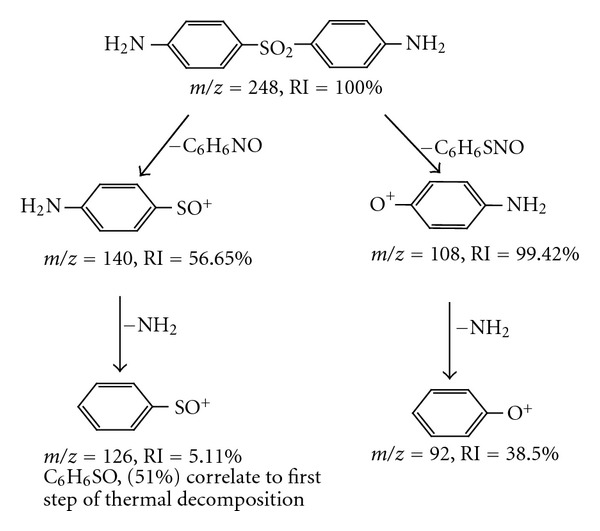
Mass spectral fragmentation pathways of Dapsone.

Mass spectrometry is used to elucidate the structure of a compound. In mass spectrometry the compound is ionized and fragmented using the electron spray ionization technique, while for thermal analysis the term decomposition signifies the breakdown of one or more constituents of the substance into simpler atomic grouping. Dapsone has symmetrical molecule which upon decomposition gives main fragments, with m/z = 140  (RI = 56.17%), m/z = 108  (RI = 92.2%), and m/z = 126  (RI = 5.11%). Fragments at m/z = 248 (RI = 100%) represent the base peaks of DDS. The electron ionization mass spectrum of the fragmented Dapsone shows an abundance of C_6_H_6_SO  (m/z = 126, 51%) molecule, suggesting that this is a major part of the decomposition lost in thermal reaction. The correlation was presented in Scheme 2.

### 3.4. Application of Differential Scanning Calorimetry for Purity Determination of Dapsone, Dimethyl Sulfone, and Topiramate in Drug Substances

The determination of purity is based on the assumption that impurities will depress that the melting point of a pure material whose melting is characterized by a melting point (*T*_0_) and an enthalpy of fusion (Δ*H*_*f*_). The melting transitions of a pure 100% crystalline material should be infinitely sharp, but impurities or defects in the crystal structure will broaden the melting range and lower the final melting point to a temperature lower than *T*_0_ [53]. Purity determination is officially listed in the British and United States Pharmacopoeias in general chapter on thermal analysis [14, 15]. The effects of impurities on *T*_0_ of DDS, MSM, and TOP were determined by DSC method based on the van't Hoff equation:

(8) $$\begin{array}{cc} T_{s}=T_{o}-\;RT_{0}^{2}\cdot \frac{1}{\Delta H_{f}}\cdot F, & \end{array}$$

where *T_s_* is the sample peak at temperature (*K*), *T*_0_ is the melting point of pure component (*K*), *R* is the gas constant, *X* is the concentration of impurity (grams fraction), Δ*H*_*f*_ is heat of fusion of pure component (J  mg^−1^), and *F* is the fraction of sample melted at *T_s_*_._

The DSC curves allowed determination of the melting points and the degrees of purity of the drugs. The results obtained by the official volumetric method, manufacturer GC method, and official HPLC-RI method for DDS, MSM, and TOP, respectively, afforded values similar to those found by DSC (Table 4). Comparison of the data on the studied drugs revealed the importance of the DSC technique for quality control of bioactive drugs. The melting points obtained by DSC revealed the precision of the technique in yielding these thermal parameters, as the RSD less than 2% (*n* = 5). This justifies the use of DSC as a routine technique for identification of drugs designed for pharmaceutical use, through the melting point.

### 3.5. Application of Differential Scanning Calorimetry for Determination of Dapsone, Dimethyl Sulfone, and Topiramate in Their Pharmaceutical Formulations

**Figure 2:**
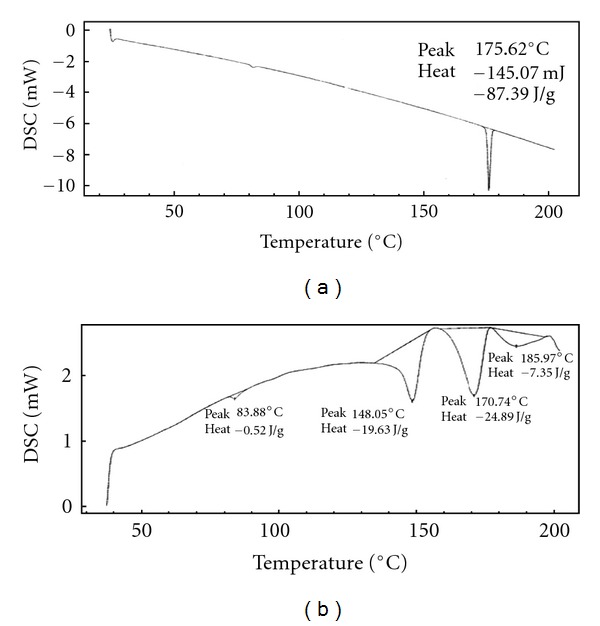
DSC profile of Dapsone drug substance (a), and Dapsone tablet (b).

**Figure 3:**
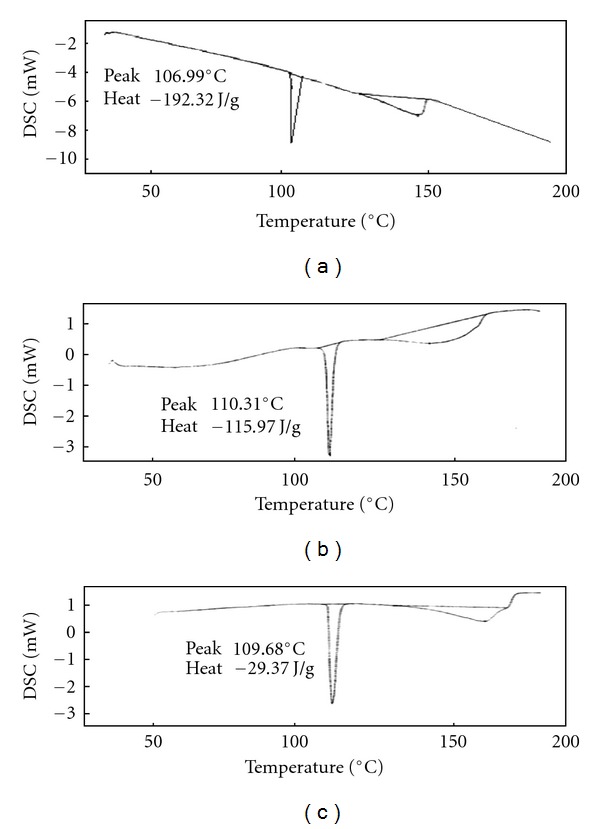
DSC profile of dimethyl sulfone drug substance (a), MSM tablet (b), and Genuphil tablet (c).

**Figure 4:**
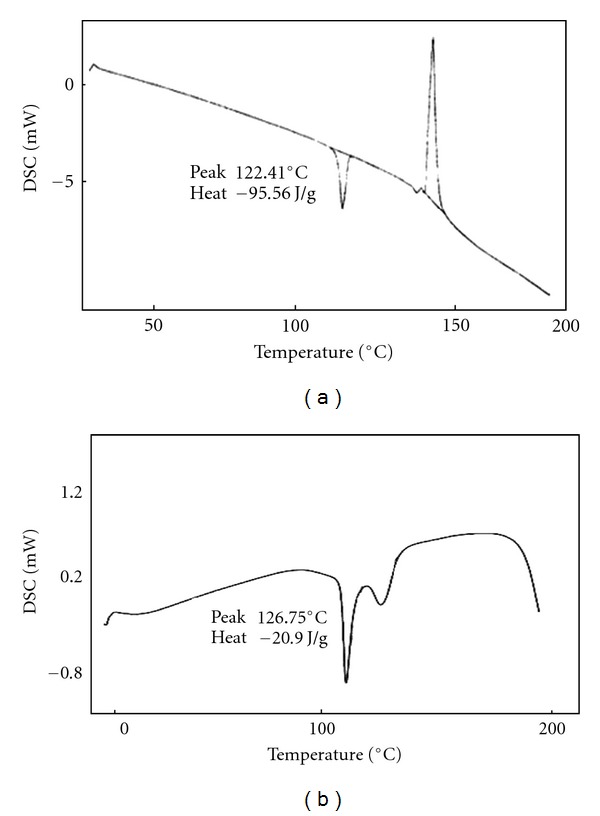
DSC profile of Topiramate drug substance (a), Topamax tablet (b).

DSC curves of DDS in drug substance and product were presented in Figure 2. Dapsone tablet (Figure 2) exhibited two shallow broaden endothermic peaks, suggesting an interaction between the drug and excipients but not necessary corresponding to incompatibility. This was attributed to drug dissolution in the melted excipient [53]. The DSC curves of MSM raw material, MSM tablet, and Genuphil tablet are presented in Figure 3. The data suggested compatibility between the drug and excipients and/or coformulated drugs. For TOP the DSC curves of raw material and Topamax tablet suggested compatibility between the drug and excipients as shown in Figure 4 this means that the excipients increase the stability of the drug. The results are stated in Table 5. The RSD is less than 5% (*n* = 5).

## 4. Conclusions

The thermal stability of DDS, MSM, and TOP using different thermal techniques (TGA/DTG, DTA, and DSC) was studied. The kinetic studies of DDS and TOP showed a thermal behavior characteristic to first order while zero for MSM. The thermal stability of the studied drugs followed the order DDS > TOP > MSM according to *E*_*a*_. The correlation between mass spectra and thermal behavior of DDS revealed correlation between the two techniques. The electron ionization mass spectrum of the fragmented Dapsone shows an abundance of C_6_H_6_SO molecule, suggesting that this is a major part of the decomposition lost in thermal reaction. The DSC method is not always appropriate for purity determination of pharmaceuticals. This method describes the determination of purity of materials greater than 98.5 mole percent purity using differential scanning calorimetry and the van't Hoff equation. The DSC data showed compatibility between the studied drugs and excipients and/or coformulated drugs. It provides a rapid method for purity determination attending a value between 98 and 102%, which is in agreement with the official pharmacopoeia. The simplicity, speed, and low operational costs of thermal analysis of pharmaceuticals justify its application in quality control.

## Figures and Tables

**Table 1:** Thermal decomposition data of TGA, DTG, and DTA curves of Dapsone, dimethyl sulfone, and Topiramate.

Drugs	TGA and DTG						DTA	
	1st reaction temperature			2nd reaction temperature			Endothermic peaks (°C)	Exothermic peaks (°C)
	Onset	End set	%wt loss	Onset	End set	%wt loss		
Dapsone	339	393.41	52.174	393.41	726	47.91	185.12 (refer to mp), 368.83, 639.34	686.34
Dimethyl sulfone	170	299.13	94.53	299.13	355	5.71	108.81 (refer to mp), 215.9	852.23
Topiramate	151	393.46	73.19	393.4	722.43	25.26	130.53 (refer to mp), 154.07	181.07, 350.80

**Table 2:** Kinetic parameters from the Arrhenius equation for Dapsone, Topiramate, and dimethyl sulfone.

Drug	Temperature range (°C)	Order of reaction	*E*_a_ (KJ moL^−1^)	ln⁡⁡*A*	*R*
Dapsone	339–393	1	485.87	88.255	0.9921
Topiramate	151–393	1	93.78	13.993	0.9957
Dimethyl sulfone	170–299	zero	51.006	11.815	0.9903

**Table 3:** Kinetic parameters obtained by the methods of Horowitz and Metzger (HM) and Coats and Redfern (CR) for Dapsone and Topiramate.

Drugs	Temperature range (°C)	*E*_a_/kJ moL^−1^		*n*		*A*·Sec^−1^
		HM	CR	HM	CR	CR
Dapsone	339–393	485	454	1	1	1.27 × 10^32^
Topiramate	151–393	136.8	144.43	1	1	7.6 × 10^8^

**Table 4:** Degree of purity and melting point of Dapsone, dimethyl sulfone, and Topiramate in drug substances by DSC, melting point apparatus, and pharmacopoeial and reported methods.

Drugs	Degree of purity%		Melting point (°C)		
	DSC*	Pharmacopoeial/reported	DSC	Mp apparatus	Pharmacopoeial/reported
Dapsone	99.66	100.29**	175.62	177	175–181
Dimethyl sulfone	99.73	99.36***	106.99	109	108–110
Topiramate	98.27	98.00****	122.41	125	122–126

*Mean of five instrumental run.

**Official BP 2010.

***Manufacturer GC method supplied by Eva Pharma, Egypt.

****Official USP 2011.

**Table 5:** Application of the proposed DSC method for determination of the claimed amount of Dapsone, dimethyl sulfone, and Topiramate in their pharmaceutical formulations.

Dosage forms	Claimed amount* % by the proposed DSC	Claimed amount % by pharmacopoeial and/or reported methods
Dapsone (50 mg of Dapsone/tab)	91.25	96.28**
MSM (100 mg MSM/tab)	95.36	94.56***
Genuphil tablet (375 mg MSM/tab)	96.33	—
Topamax (25 mg TOP/tab)	93.54	94.26****

*Mean of five instrumental run.

**Official BP 2010.

***Manufacturer GC method supplied by Eva Pharma, Egypt.

****Official USP 2012.

## References

[1] Tomecki KJ, Catalano CJ (1981). Dapsone hypersensitivity. The sulfone syndrome revisited. *Archives of Dermatology*.

[2] Richardus JH, Smith TC (1989). Increased incidence in leprosy of hypersensitivity reactions to dapsone after introduction of multidrug therapy. *Leprosy Review*.

[3] (2010). *British Pharmacopoeia*.

[4] The United States Pharmacopoeia USP 34 (2011). *The National Formulary NF 29*.

[5] Hucker HB, Ahmad PM, Miller EA, Brobyn R (1966). Metabolism of dimethyl sulphoxide to dimethyl sulphone in the rat and man. *Nature*.

[6] Murav’ev LUV, Venikova MS, Pleskovskai NG (1991). *Patologicheskaia Fiziologiia i Eksperimental’naia Terapiia*.

[7] Kocsis JJ, Harkaway S, Snyder R (1975). Biological effects of the metabolites of dimethyl sulfoxide. *Annals of the New York Academy of Sciences*.

[8] Liu JL, LI S, LI ZH, Ma H (2002). Capillary gas-chromatographic separation and determination of dimethyl sulfoxide and dimethyl sulfone. *Lihua Jianyan, Huaxue Fence*.

[9] Gillman AG, Limbird LE, Hardman JG (2006). *Goodman and Gillman’s The Pharmacological Basis of Therapeutics*.

[10] Nagaraja P, Yathirajan HS, Sunitha KR, Vasantha RA (2002). Novel methods for the rapid spectrophotometric determination of dapsone. *Analytical Letters*.

[11] Revanasiddappa HD, Manju B (2002). Spectrophotometric determination of some chemotherapeutic agents using acetyl acetone. *Drug Development and Industrial Pharmacy*.

[12] Nagaraja P, Sunitha KR, Vasantha RA, Yathirajan HS (2001). A sensitive method for the spectrophotometric determination of dapsone. *Indian Drugs*.

[13] Revanasiddappa HD, Manju B (2001). A spectrophotometric method for the determination of metoclopramide HCl and dapsone. *Journal of Pharmaceutical and Biomedical Analysis*.

[14] Sastry CSP, Srinivas KR, Prasad KMMK (1996). Spectrophotometric determination of bio-active compounds in commercial samples with nitrous acid and cresyl fast violet acetate. *Analytical Letters*.

[15] Toral MI, Tassara A, Soto C, Richter P (2003). Simultaneous determination of dapsone and pyrimethamine by derivative spectrophotometry in pharmaceutical formulations. *Journal of AOAC International*.

[16] Evgen’ev MI, Garmonov SY, Pogorel’tsev VI, Shakirova EF (1999). Determination of 4,4′-diaminodiphenyl sulfone and its derivatives in biological samples by spectrophotometry and chromatography. *Journal of Analytical Chemistry*.

[17] Shetty KT, Naik PM, Mahadevan PR (1990). A specific colorimetric assay for dapsone in biological fluids. *Indian Journal of Clinical Biochemistry*.

[18] Shoukrallah I, Sakla A, Wintersteiger R (1990). Spectrophotometric determination of dapsone by using 9-chloroacridine as a chromogenic reagent. *Pharmazie*.

[19] Tawada JC, Midio AF (1989). The determination of dapsone in plasma and urine. *Revista de Farmacia e Bioquimica da Universidade de Sao Paulo*.

[20] Ma L, Tang B, Chu C (2002). Spectrofluorimetric study of the *β*-cyclodextrin–dapsone–linear alcohol supramolecular system and determination of dapsone. *Analytica Chimica Acta*.

[21] Shukrallah IZF, Sakla AB (1988). The use of the protou magnetic pesonance (PMR) spectroscopy in the quantitative determination of dapsone in the bulk and tablets. *Spectroscopy Letters*.

[22] Oelschlager H, Modrack G (1986). Analysis of drugs by polarographic methods, XXVI: polarographic determination (DPP) of the antileprosy agent diaphenylsulfone. *Archiv der Pharmazie*.

[23] Manisankar P, Sarpudeen A, Viswanathan S (2001). Electroanalysis of dapsone, an anti-leprotic drug. *Journal of Pharmaceutical and Biomedical Analysis*.

[24] Lemnge MM, Roenn A, Flachs H, Bygbjerb IC (1993). Simultaneous determination of dapsone, monoacetyldapsone and pyrimethamine in whole blood and plasma by high-performance liquid chromatography. *Journal of Chromatography B*.

[25] Moncrieff J (1994). Determination of dapsone in serum and saliva using reversed-phase high-performance liquid chromatography with ultraviolet or electrochemical detection. *Journal of Chromatography B*.

[26] Queiroz RHC, Dreossi SAC, Carvalho D (1997). A rapid, specific, and sensitive method for the determination of acetylation phenotype using dapsone. *Journal of Analytical Toxicology*.

[27] Tracqui A, Gutbub AM, Kintz P, Mangin P (1995). A Case of Acute Dapsone Poisoning: Toxicological Data and Review of the Literature. *Journal of Analytical Toxicology*.

[28] Ronn AM, Lemnge MM, Angelo HR, Bygbjerg IC (1995). High-performance liquid chromatography determination of dapsone, monoacetyldapsone, and pyrimethamine in filter paper blood spots. *Therapeutic Drug Monitoring*.

[29] Takeuchi A, Yamamoto S, Narai R (2010). Determination of dimethyl sulfoxide and dimethyl sulfone in urine by gas chromatography-mass spectrometry after preparation using 2,2-dimethoxypropane. *Biomedical Chromatography*.

[30] Micheel AP, Ko CY, Guh HY (1998). Ion chromatography method and validation for the determination of sulfate and sulfamate ions in topiramate drug substance and finished product. *Journal of Chromatography B*.

[31] Styslo-Zalasik M, Li W (2005). Determination of topiramate and its degradation product in liquid oral solutions by high performance liquid chromatography with a chemiluminescent nitrogen detector. *Journal of Pharmaceutical and Biomedical Analysis*.

[32] Biro A, Pergel E, Arvai G (2006). High-performance liquid chromatographic study of topiramate and its impurities. *Chromatographia*.

[33] Bahrami G, Mohammadi B (2007). A novel high sensitivity HPLC assay for topiramate, using 4-chloro-7-nitrobenzofurazan as pre-column fluorescence derivatizing agent. *Journal of Chromatography B*.

[34] Contin M, Riva R, Albani F, Baruzzi A (2001). Simple and rapid liquid chromatographic-turbo ion spray mass spectrometric determination of topiramate in human plasma. *Journal of Chromatography B*.

[35] Bahrami G, Mirzaeei S, Mohammadi B, Kiani A (2005). High performance liquid chromatographic determination of topiramate in human serum using UV detection. *Journal of Chromatography B*.

[36] Tang PH, Miles MV, Glauser TA (2000). An improved gas chromatography assay for topiramate monitoring in pediatric patients. *Therapeutic Drug Monitoring*.

[37] Holland ML, Uetz JA, Ng KT (1988). Automated capillary gas chromatographic assay using flame ionization detection for the determination of topiramate in plasma. *Journal of Chromatography*.

[38] Riffitts JM, Gisclon LG, Stubbs RJ, Palmer ME (1999). A capillary gas chromatographic assay with nitrogen phosphorus detection for the quantification of topiramate in human plasma, urine and whole blood. *Journal of Pharmaceutical and Biomedical Analysis*.

[39] Chen S, M. Carvey P (2001). *Rapid Communications in Mass Spectrometry*.

[40] Berry DJ, Patsalos PN (2000). Comparison of topiramate concentrations in plasma and serum by fluorescence polarization immunoassay. *Therapeutic Drug Monitoring*.

[41] Wendlandt WW, Collins LW (1974). The identification of non-prescription internal analgesics by thermal analysis. *Analytica Chimica Acta*.

[42] Valladao DMS, De Oliveira LCS, Zuanon Netto J, Ionashiro M (1996). Thermal decomposition of some diuretic agents. *Journal of Thermal Analysis*.

[43] Gupchup G, Alexander K, Dollimore D (1992). The use of thermal analysis and mass spectrometry to study the solid state behavior in pharmaceutical tablet mixtures. *Thermochimica Acta*.

[44] Macêdo RO, de Souza AG, Macêdo AMC (1997). Application of thermogravimetry in the quality control of mebendazole. *Journal of Thermal Analysis*.

[45] Wunderlich B (1990). *Thermal Analysis*.

[46] Dollimore D (1999). A breath of fresh air. *Thermochimica Acta*.

[47] Horowitz HH, Metzger G (1963). A new analysis of thermogravimetric traces. *Analytical Chemistry*.

[48] Singh P, Premkumar L, Mehrotra R, Kandpal HC, Bakhshi AK (2008). Evaluation of thermal stability of indinavir sulphate using diffuse reflectance infrared spectroscopy. *Journal of Pharmaceutical and Biomedical Analysis*.

[49] Menen D, El-Ries M, Alexender KS, Rigo A, Dollimere D (2002). A Thermal Analysis Study of the Decomposition of Hydrochlorthiazide. *Instrumentation Science and Technology*.

[50] Coats AW, Redfern JP (1964). Kinetic parameters from thermogravimetric data. *Nature*.

[51] Brown ME (1988). *Introduction to Thermal Analysis*.

[52] Dollimore D, Tong P, Alexander KS (1996). The kinetic interpretation of the decomposition of calcium carbonate by use of relationships other than the Arrhenius equation. *Thermochimica Acta*.

[53] Araújo A, Bezerra MS, Storpirtis S, Matos J (2010). Determination of the melting temperature, heat of fusion, and purity analysis of different samples of zidovudine (AZT) using DSC. *Brazilian Journal of Pharmaceutical Sciences*.

